# Development and temporal validation of an explainable machine learning model for predicting in-hospital diabetic foot ulcer in patients with type 2 diabetes using inflammatory–nutritional composite indices

**DOI:** 10.3389/fmed.2026.1938429

**Published:** 2026-10-23

**Authors:** Xue Wang, Chao Li, Yanan Tuo, Bo Zhao, Ayiding Xiahati, Lu Fan, Paerhati Wahafu

**Affiliations:** The Sixth Affiliated Hospital of Xinjiang Medical University, Urumqi, Xinjiang Uygur Autonomous Region, China

**Keywords:** diabetic foot ulcer, explainable artificial intelligence, inflammatory biomarkers, machine learning, nutritional status, shapley additive explanations, temporal external validation, type 2 diabetes mellitus

## Abstract

**Objective:**

This study aimed to develop and temporally validate explainable machine learning models for predicting in-hospital new-onset DFU in hospitalized patients with type 2 diabetes mellitus (T2DM) using routinely available admission variables and inflammatory–nutritional composite indices.

**Methods:**

This retrospective prediction-model study enrolled 550 consecutive hospitalized patients with T2DM admitted between January 2022 and December 2024. Patients admitted during 2022–2023 (*n* = 400) constituted the development cohort, whereas those admitted in 2024 (*n* = 150) served as an independent temporal validation cohort. Candidate predictors included demographic characteristics, diabetes duration, routine laboratory biomarkers, ankle–brachial index (ABI), and inflammatory–nutritional composite indices, including neutrophil percentage-to-albumin ratio (NPAR), albumin-to-neutrophil-to-lymphocyte ratio (ANLR), hemoglobin–albumin–lymphocyte–platelet score (HALP), systemic immune-inflammation index (SII), and C-reactive protein-to-albumin ratio (CRP/ALB). Candidate variables were screened using univariable logistic regression followed by least absolute shrinkage and selection operator (LASSO) regression. Logistic regression, random forest, LightGBM, and XGBoost models were developed and evaluated using the area under the receiver operating characteristic curve (AUC), average precision (AP), sensitivity, specificity, F1-score, Brier score, calibration analysis, and decision curve analysis (DCA). Model interpretability was assessed using SHapley Additive exPlanations (SHAP).

**Results:**

During hospitalization, 66 patients (12.0%) developed DFU. Eleven predictors were retained after feature selection, including age, diabetes duration, neutrophil percentage, C-reactive protein, high-density lipoprotein cholesterol, glycated hemoglobin, ABI, NPAR, ANLR, HALP, and SII. In the temporal validation cohort, logistic regression had the highest observed discrimination (AUC = 0.976; bootstrap 95% CI, 0.936–0.992), with a sensitivity of 0.944, specificity of 0.932, and F1-score of 0.773 at the prespecified threshold; however, its Hosmer–Lemeshow test indicated evidence of lack of fit (*P* = 0.001). LightGBM had the lowest Brier score (0.064), whereas XGBoost achieved an AUC of 0.945, a Brier score of 0.067, and a Hosmer–Lemeshow *P* value of 0.350. At the prespecified thresholds, LightGBM and XGBoost sensitivities were only 0.056 and 0.389, respectively. SHAP analysis identified ANLR, glycated hemoglobin, HALP, age, and SII as the five most influential XGBoost predictors. Because the temporal validation cohort contained only 18 DFU events, these performance estimates should be interpreted cautiously.

**Conclusion:**

Admission-based machine learning models showed high observed discrimination for in-hospital DFU risk stratification, but no model was uniformly superior across discrimination, calibration, and threshold-dependent sensitivity. The small number of validation events, possible redundancy among mathematically overlapping predictors, omitted established foot-risk variables, and the low sensitivity of LightGBM and XGBoost at their prespecified thresholds preclude direct use as screening tools. Prospective multicenter validation, formal assessment of predictor redundancy, threshold recalibration, and clinical-impact evaluation are required before implementation.

## 1 Introduction

Diabetic foot ulcer (DFU) is one of the most devastating chronic complications of diabetes, commonly driven by a combination of peripheral neuropathy, peripheral arterial disease, foot deformity, infection, and repetitive mechanical stress. Recent reviews indicate that DFU not only heals slowly and recurs frequently but is also closely associated with an increased risk of infection, amputation, and mortality (1). The 2023 guidelines of the International Working Group on the Diabetic Foot (IWGDF) emphasize that risk-stratified foot screening, patient education, offloading, revascularisation assessment, and infection management are essential to reduce the burden of ulcer development and recurrence (2–4). Hence, early identification of high-risk individuals using readily available information during hospitalisation represents a critical step in the pre-emptive prevention and management of DFU.

Traditional DFU risk assessment has largely relied on classical risk factors, including diabetes duration, neuropathy, prior foot ulcers, foot deformities, peripheral arterial disease, and glycaemic control (5–7). However, hospitalised patients frequently present with acute or chronic inflammatory responses, diminished nutritional reserves, and vascular dysfunction; relying solely on individual clinical indicators may be insufficient to capture the risk of in-hospital new-onset DFU. Inflammatory-nutritional composite indices derived from routine blood and biochemical tests—such as the neutrophil-to-lymphocyte ratio, platelet-to-lymphocyte ratio, systemic immune-inflammation index (SII), C-reactive protein-to-albumin ratio, and haemoglobin-albumin-lymphocyte-platelet (HALP) score—have attracted growing attention because they are inexpensive, readily available, and reproducible (8–10). These composite measures reflect host status from three dimensions—immune activation, nutritional reserve, and systemic inflammation—and may provide incremental information for DFU risk prediction beyond traditional single parameters.

Machine learning (ML) methods, which are capable of capturing non-linear relationships and variable interactions, have been applied to predict DFU occurrence, amputation, wound healing, and complication risks (11, 12). Nevertheless, current studies in this field share three major shortcomings. First, random splitting of training and test sets may cause temporal information leakage, leading to over-optimistic estimates of model performance in future patients. Second, limited model interpretability hinders clinical adoption. Third, some investigations emphasise discrimination while neglecting calibration and net clinical benefit. Recently, the TRIPOD + AI and PROBAST + AI statements have stressed that prediction model studies should transparently report data sources, validation strategies, model development processes, calibration, interpretability, and potential biases.

Against this backdrop, the present study enrolled hospitalised patients with type 2 diabetes from 2022 to 2024, using cases from 2022 to 2023 for model development and those from 2024 for temporal external validation. By combining univariable screening, LASSO-based variable compression, comparisons of four model types, calibration curves, decision curve analysis, and SHAP (SHapley Additive exPlanations) interpretation, we aim to develop and evaluate a DFU risk prediction model based on inflammatory–nutritional composite indices.

## 2 Methods

### 2.1 Study design and study population

This retrospective prediction-model study was conducted at the Sixth Affiliated Hospital of Xinjiang Medical University in accordance with the TRIPOD + AI statement (13) and the Declaration of Helsinki. The study was approved by the Ethics Committee of the Sixth Affiliated Hospital of Xinjiang Medical University (Approval No. LFYLLSC20220811-12), and the requirement for written informed consent was waived because anonymized retrospective clinical data were used. Consecutive patients with type 2 diabetes mellitus (T2DM) hospitalized between January 2022 and December 2024 were retrospectively screened.

The inclusion criteria were as follows: (1) diagnosis of T2DM according to established diagnostic criteria; (2) absence of active diabetic foot ulcer (DFU) at admission; and (3) availability of routine laboratory examinations within 48 h after admission. Patients were excluded if they (1) had missing outcome data or key laboratory variables; (2) presented with severe trauma, active sepsis, or advanced malignancy at admission; or (3) had repeated hospitalization records that could not be identified as independent observations.

A total of 550 eligible patients were included in the final analysis. To simulate real-world clinical deployment and minimize temporal information leakage, patients admitted during 2022–2023 were assigned to the development cohort (*n* = 400), whereas patients admitted during 2024 constituted an independent temporal validation cohort (*n* = 150, Figure 1).

**Figure 1:**
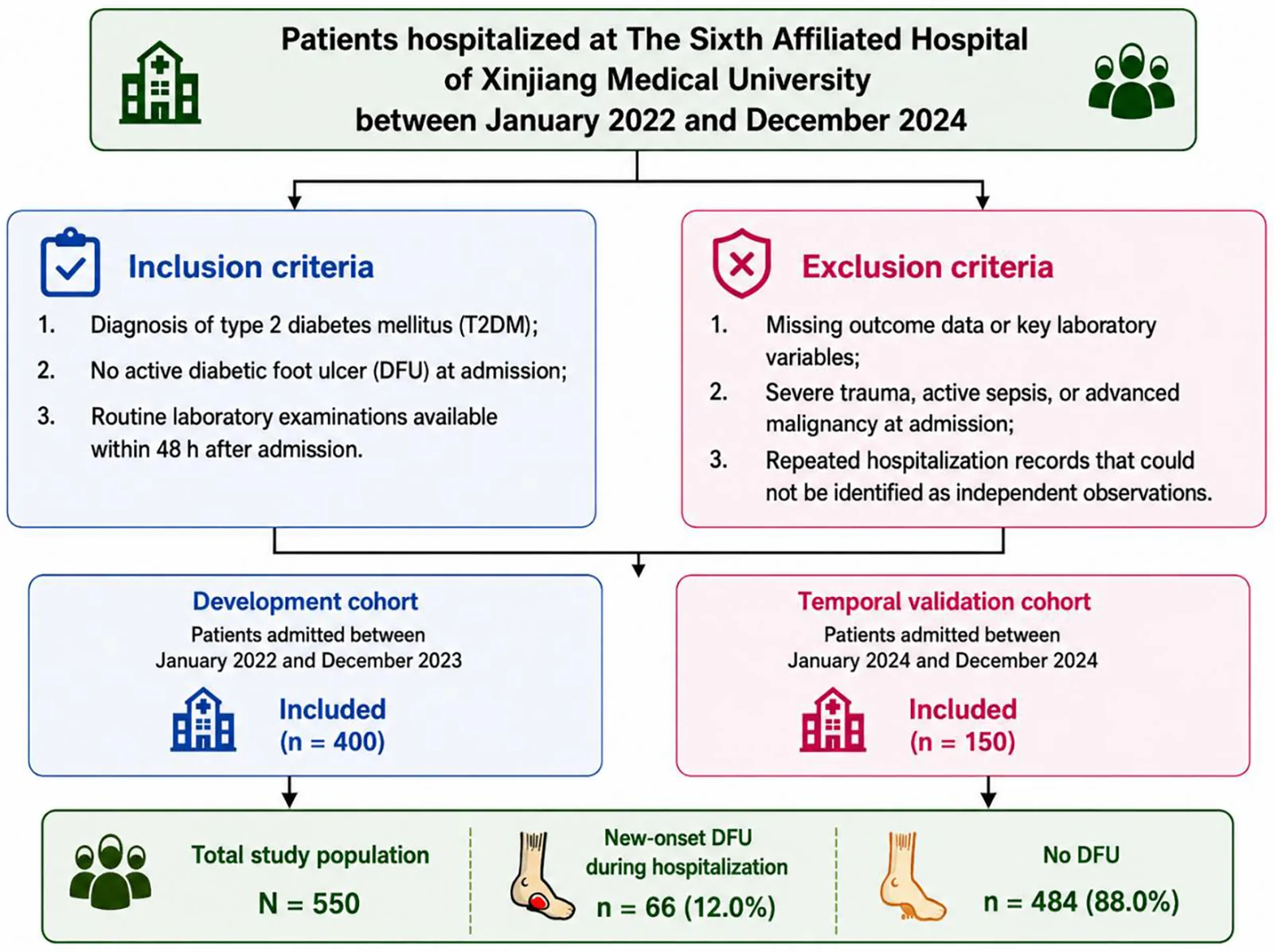
Flowchart of patient screening.

### 2.2 Outcome definition

The primary outcome was new-onset DFU during hospitalization after admission; patients with active DFU at admission were excluded. DFU was defined according to the International Working Group on the Diabetic Foot (IWGDF) guidelines as a full-thickness lesion involving the skin and underlying tissues below the ankle in patients with diabetes, with or without evidence of infection or ischemia (14). Outcome determination was based on electronic medical records, discharge diagnoses, nursing documentation, and specialist foot examinations. Admission laboratory predictors were obtained within 48 h after admission. Exact admission-to-DFU time, hospital length of stay, and blinding of outcome assessment to predictor information were not available for this retrospective analysis.

### 2.3 Candidate predictors

Candidate predictors were selected based on clinical relevance and previous literature and included demographic characteristics, diabetes history, routine laboratory biomarkers, vascular assessment, and inflammatory–nutritional composite indices.

Demographic and clinical variables included age, sex, diabetes duration, smoking history, alcohol consumption, hypertension, systolic blood pressure, and diastolic blood pressure.

Laboratory variables included neutrophil percentage, lymphocyte count, platelet count, hemoglobin, serum albumin, C-reactive protein (CRP), high-density lipoprotein cholesterol (HDL-C), glycated hemoglobin (HbA1c), and serum creatinine.

Peripheral arterial perfusion was assessed using the ankle–brachial index (ABI). Continuous variables were retained in their original form without categorization whenever possible to preserve statistical information.

### 2.4 Inflammatory–nutritional composite indices

The composite indices were calculated from admission laboratory data as follows: NPAR = neutrophil percentage (%)/albumin (g/L); ANLR = albumin (g/L)/[(neutrophil percentage/100)/Lymphocyte count (×10^9^/L)]; HALP = hemoglobin (g/L) × albumin (g/L) × lymphocyte count (×10^9^/L)/platelet count (×10^9^/L); SII = platelet count × neutrophil percentage (%)/lymphocyte count;and CRP/ALB = CRP (mg/L)/albumin (g/L) (15, 16). These indices were intended to summarize inflammatory activation, circulating immune-cell composition, and nutritional reserve.

### 2.5 Feature selection, collinearity assessment, and model development

Feature selection was performed exclusively in the development cohort. Univariable binary logistic regression was first conducted, and variables with *P* < 0.20 were retained to reduce the risk of prematurely excluding weak but potentially informative predictors. The least absolute shrinkage and selection operator (LASSO) was then fitted with eight-fold stratified cross-validation to determine the penalty parameter. Predictors with non-zero coefficients were retained for model development. Pearson correlation coefficients were calculated for the selected predictors and visualized in a correlation heatmap. Because several composite indices share mathematical components, the heatmap was interpreted descriptively; neither pairwise correlation nor LASSO was considered sufficient to establish independence among retained predictors.

Four models were developed using the selected predictors: logistic regression, random forest, LightGBM, and XGBoost. Eight-fold stratified cross-validation was used within the development cohort to evaluate internal stability. Class imbalance was addressed through model-specific weighting or sampling-related parameters. The classification threshold selected in the development cohort was fixed before application to cross-validation and temporal validation data, thereby avoiding threshold optimization in the validation cohort (Figure 2).

**Figure 2:**
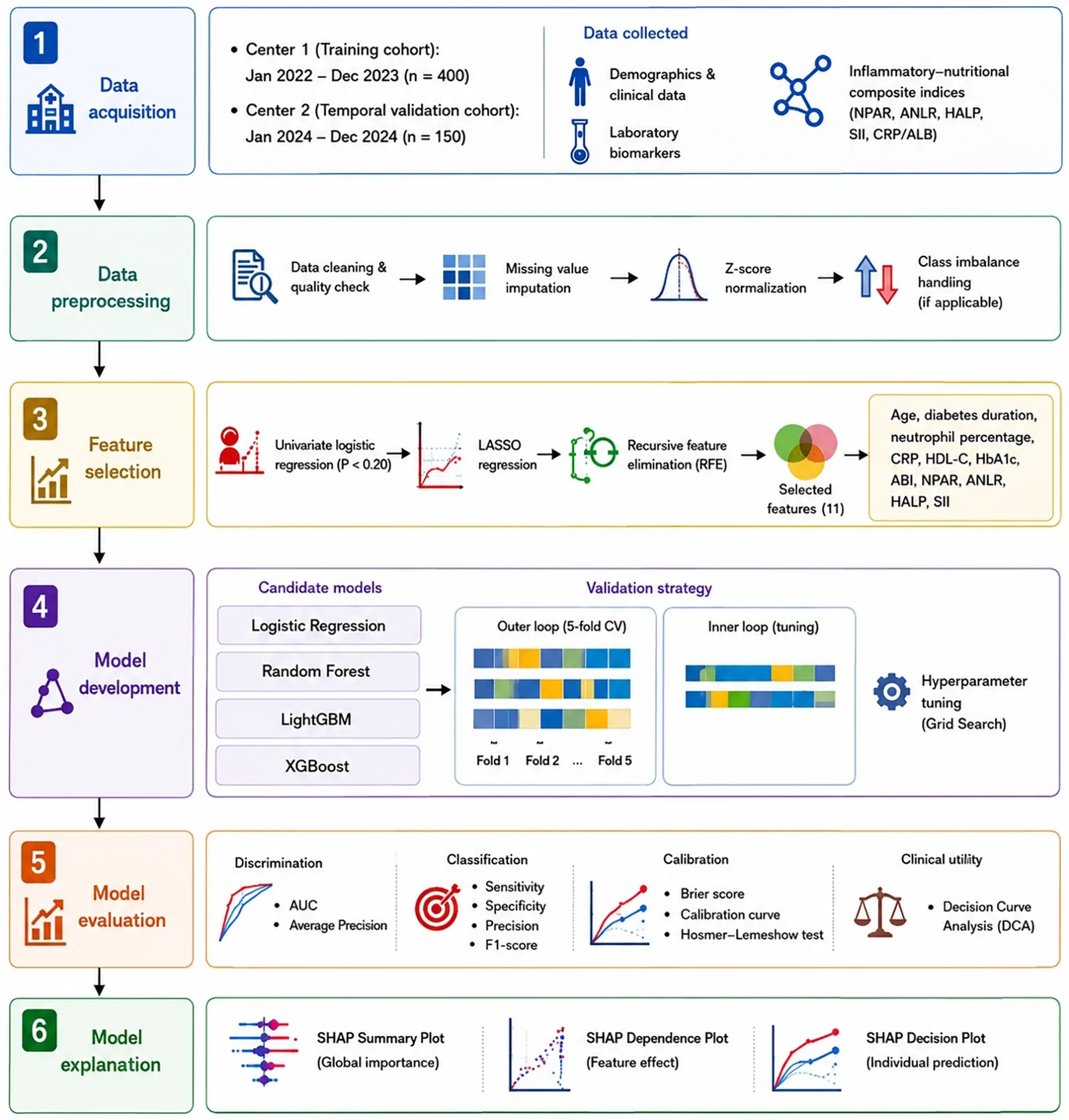
Machine learning workflow. ABI, ankle-brachial index; AP, average precision; DCA, decision curve analysis; DFU, diabetic foot ulcer; PR, precision-recall; ROC, receiver operating characteristic; SHAP, SHapley Additive exPlanations.

### 2.6 Model evaluation and interpretation

Discrimination was evaluated using the area under the receiver operating characteristic curve (AUC) and average precision (AP). Sensitivity, specificity, precision, and F1 score were calculated at the prespecified threshold. Calibration was evaluated using the Brier score, calibration plots, and the Hosmer-Lemeshow goodness-of-fit test. Clinical utility was assessed using decision curve analysis, which compared each model with treat-all and treat-none strategies across a range of threshold probabilities (17).

TreeExplainer was used to calculate SHAP values for the XGBoost model. Global interpretation was performed using mean absolute SHAP values and a beeswarm plot, and partial dependence analysis was used to illustrate potentially nonlinear relationships between key predictors and predicted DFU risk (18).

### 2.7 Statistical analysis

Continuous variables are presented as mean ± standard deviation (SD), whereas categorical variables are presented as frequencies and percentages. Between-group comparisons were performed using the independent-samples t-test for continuous variables and the *χ*^2^ test or Fisher's exact test for categorical variables, as appropriate. All statistical analyses and machine learning model development were performed using Python (version 3.10). The following major libraries were used: pandas (version 1.5.3), numpy (version 1.24.3), scikit-learn (version 1.2.2) for logistic regression and preprocessing, LightGBM (version 3.3.5), XGBoost (version 1.7.5), and shap (version 0.41.0) for model interpretation. Visualization was performed using matplotlib (version 3.7.1) and seaborn (version 0.12.2). All statistical tests were two-sided, and a *P*-value <0.05 was considered statistically significant. Missing data were handled using complete-case analysis, as the proportion of missing values for each candidate predictor was <5%. No imputation was performed. Patients with missing outcome data or missing key laboratory variables were excluded at the screening stage (see Inclusion/Exclusion criteria).

### 2.8 Ethics statement

This retrospective study was approved by the Ethics Committee of the Sixth Affiliated Hospital of Xinjiang Medical University (Approval No. LFYLLSC20220811-12). The requirement for written informed consent was waived owing to the retrospective nature of the study and the use of anonymized clinical data, in accordance with institutional regulations and the Declaration of Helsinki.

## 3 Results

### 3.1 Study population and baseline characteristics

A total of 550 hospitalized patients with T2DM were included: 400 in the development cohort and 150 in the temporal validation cohort. During hospitalization, 66 patients developed new-onset DFU, corresponding to an overall event rate of 12.0%. The development and temporal validation cohorts included 48 and 18 events, respectively, and both cohorts had an event rate of 12.0%. Baseline demographic, clinical, laboratory, vascular, and composite-index characteristics were broadly comparable between the cohorts (Table 1).

**Table 1:** Baseline characteristics of the development and temporal validation cohorts.

Characteristic	Development cohort (*n* = 400)	Temporal validation cohort (*n* = 150)	*P*-value
Age, years	62.23 ± 9.52	60.73 ± 10.14	0.117
Male sex, *n* (%)	219 (54.8%)	73 (48.7%)	0.239
Diabetes duration, years	8.40 ± 4.79	9.21 ± 4.95	0.089
Smoking history, *n* (%)	117 (29.2%)	44 (29.3%)	1.000
Alcohol use, *n* (%)	104 (26.0%)	36 (24.0%)	0.712
Hypertension, *n* (%)	243 (60.8%)	86 (57.3%)	0.529
Dyslipidemia, *n* (%)	185 (46.2%)	79 (52.7%)	0.213
Neutrophils, %	60.45 ± 10.43	58.61 ± 9.86	0.057
Lymphocyte count, ×10^9^/L	1.96 ± 0.78	1.92 ± 0.76	0.526
Platelet count, ×10^9^/L	219.48 ± 57.92	217.27 ± 60.32	0.699
Hemoglobin, g/L	130.46 ± 19.30	127.19 ± 19.47	0.080
Albumin, g/L	37.53 ± 5.10	37.98 ± 5.39	0.371
CRP, mg/L	3.27 ± 2.55	3.10 ± 2.13	0.415
Creatinine, micromol/L	78.80 ± 31.12	81.82 ± 29.15	0.290
Urea, mmol/L	6.06 ± 2.48	5.89 ± 2.43	0.479
Uric acid, micromol/L	356.71 ± 99.25	346.05 ± 104.29	0.280
Total cholesterol, mmol/L	4.57 ± 1.22	4.66 ± 1.18	0.451
Triglycerides, mmol/L	1.53 ± 0.81	1.50 ± 0.95	0.721
HDL-C, mmol/L	1.10 ± 0.28	1.08 ± 0.27	0.385
LDL-C, mmol/L	2.80 ± 0.85	2.83 ± 0.85	0.792
Fasting plasma glucose, mmol/L	7.44 ± 2.34	7.28 ± 2.14	0.427
HbA1c, %	7.55 ± 1.38	7.42 ± 1.39	0.333
ABI	1.00 ± 0.19	1.00 ± 0.19	0.844
NPAR	1.64 ± 0.37	1.58 ± 0.40	0.120
ANLR	125.74 ± 58.33	127.26 ± 58.72	0.787
HALP	48.64 ± 30.83	46.39 ± 26.14	0.395
CRP/ALB	0.09 ± 0.07	0.08 ± 0.06	0.274
SII	86,326.55 ± 66,487.45	83,666.98 ± 57,058.89	0.642
New-onset DFU, *n* (%)	48 (12.0%)	18 (12.0%)	1.000

Data are mean ± standard deviation or *n* (%).

ABI, ankle-brachial index; ANLR, albumin-to-neutrophil-to-lymphocyte ratio; CRP, C-reactive protein; CRP/ALB, C-reactive protein-to-albumin ratio; DFU, diabetic foot ulcer; HALP, hemoglobin-albumin-lymphocyte-platelet score; HDL-C, high-density lipoprotein cholesterol; LDL-C, low-density lipoprotein cholesterol; NPAR, neutrophil percentage-to-albumin ratio; SII, systemic immune-inflammation index.

### 3.2 Feature selection

Thirteen variables met the prespecified *P* < 0.20 criterion in univariable logistic regression and were entered into LASSO regression. Eleven predictors with non-zero coefficients were retained: age, diabetes duration, neutrophil percentage, CRP, HDL-C, HbA1c, ABI, NPAR, ANLR, HALP, and SII. Lymphocyte count and albumin were reduced to zero by LASSO and excluded from model development. CRP/ALB did not meet the univariable screening criterion and was not forcibly included. In the multivariable logistic regression model, age, diabetes duration, HbA1c, and ANLR remained statistically significant (Table 2). The LASSO coefficient paths, cross-validation curve, multivariable logistic regression forest plot, and predictor correlation matrix are shown in Figure 3. Because the retained predictor set included neutrophil percentage together with composite indices derived from overlapping neutrophil, lymphocyte, albumin, hemoglobin, and platelet components, these predictors should not be interpreted as statistically independent; Figure 3D was used as a descriptive correlation assessment rather than definitive evidence against multicollinearity.

**Table 2:** Feature selection and multivariable logistic regression results in the development cohort.

Predictor	Univariable OR (95% CI)	*P*-value	LASSO coefficient	Selected	Adjusted OR (95% CI)	*P*-value
Age	1.31 (0.97–1.77)	0.082	1.220	Yes	3.10 (1.69–5.70)	<0.001
Diabetes duration	1.45 (1.07–1.97)	0.016	0.809	Yes	2.25 (1.31–3.88)	0.003
Neutrophil percentage	1.56 (1.14–2.12)	0.005	0.247	Yes	1.60 (0.69–3.72)	0.277
CRP	0.77 (0.52–1.14)	0.188	−0.291	Yes	0.65 (0.37–1.13)	0.126
HDL-C	0.77 (0.57–1.05)	0.101	−0.209	Yes	0.78 (0.46–1.34)	0.375
HbA1c	2.65 (1.87–3.76)	<0.001	2.442	Yes	10.88 (4.51–26.23)	<0.001
ABI	0.76 (0.57–1.03)	0.077	−0.287	Yes	0.65 (0.38–1.13)	0.126
NPAR	1.93 (1.43–2.62)	<0.001	0.102	Yes	1.05 (0.49–2.24)	0.907
ANLR	0.08 (0.04–0.16)	<0.001	−5.039	Yes	0.01 (0.00–0.06)	<0.001
HALP	0.11 (0.05–0.22)	<0.001	−0.158	Yes	0.83 (0.25–2.75)	0.759
SII	2.61 (2.00–3.41)	<0.001	0.141	Yes	1.04 (0.47–2.29)	0.920
Lymphocyte count	0.19 (0.12–0.30)	<0.001	0.000	No	–	–
Albumin	0.59 (0.43–0.81)	0.001	0.000	No	–	–

LASSO coefficients were estimated using standardized predictors.

ABI, ankle-brachial index; ANLR, albumin-to-neutrophil-to-lymphocyte ratio; CI, confidence interval; CRP, C-reactive protein; HALP, hemoglobin-albumin-lymphocyte-platelet score; HDL-C, high-density lipoprotein cholesterol; NPAR, neutrophil percentage-to-albumin ratio; OR, odds ratio; SII, systemic immune-inflammation index.

**Figure 3:**
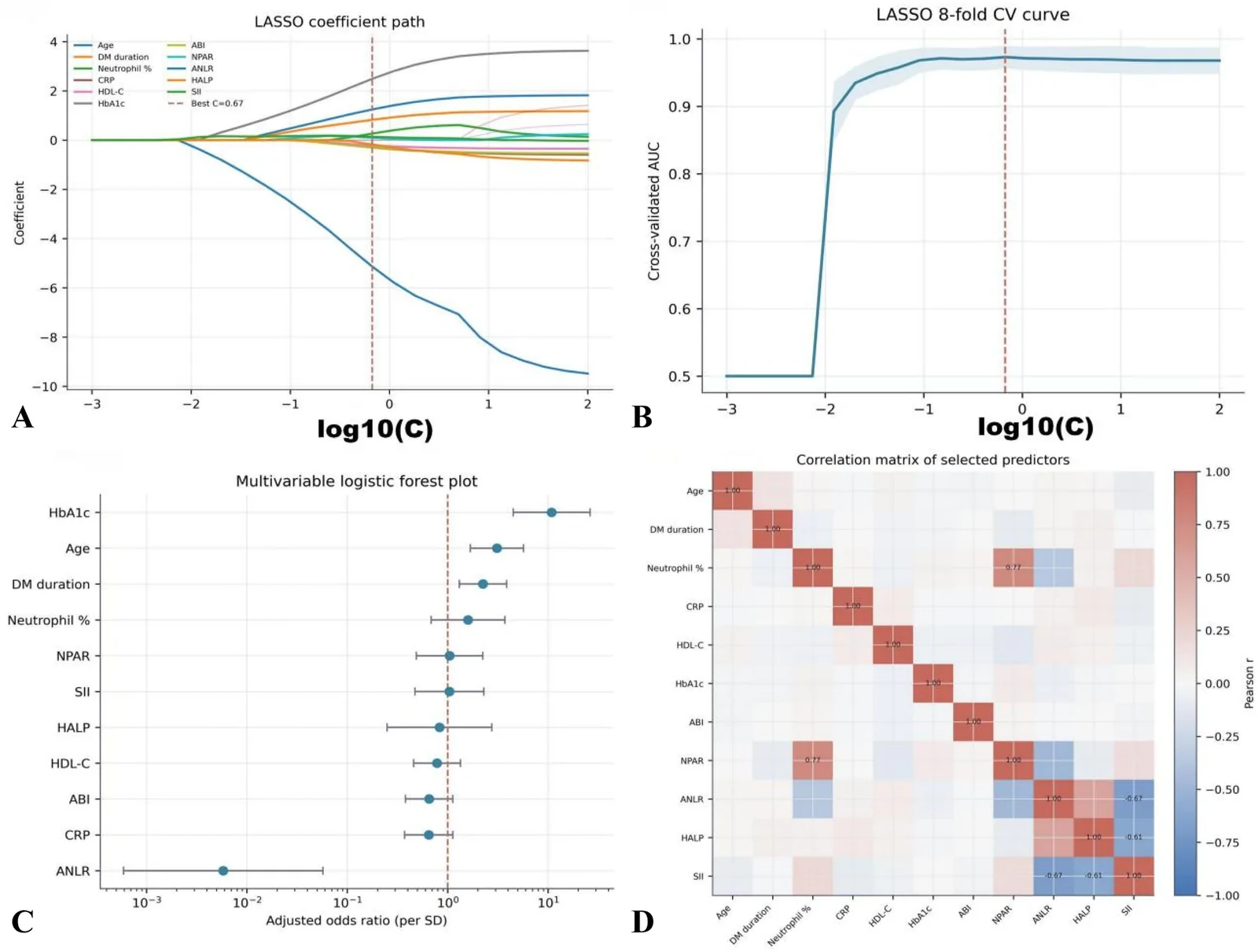
Feature selection and collinearity assessment. **(A)** LASSO coefficient path; **(B)** eight-fold cross-validation curve; **(C)** multivariable logistic regression forest plot; and **(D)** pearson correlation matrix of the selected predictors.

### 3.3 Model performance in the temporal validation cohort

The performance of the four models is summarized in Table 3 and Figure 4. Logistic regression achieved the highest observed discrimination, with an AUC of 0.976 (bootstrap 95% CI, 0.936–0.992) and AP of 0.762. At the threshold of 0.593 selected in the development cohort, sensitivity was 0.944, specificity was 0.932, precision was 0.654, and the F1 score was 0.773. The model identified 17 of the 18 patients who developed DFU and generated nine false-positive predictions. Because the temporal validation cohort contained only 18 positive events, these point estimates are sensitive to the classification of a small number of patients and should be interpreted cautiously.

**Table 3:** Predictive performance of the four models in the temporal validation cohort.

Model	AUC	AP	Sensitivity	Specificity	Precision	F1 score	Brier score	HL *P*-value	Threshold	TP	FP	TN	FN
Logistic regression	0.976	0.762	0.944	0.932	0.654	0.773	0.066	0.001	0.593	17	9	123	1
Random forest	0.943	0.599	0.889	0.879	0.500	0.640	0.077	0.109	0.472	16	16	116	2
LightGBM	0.942	0.612	0.056	0.992	0.500	0.100	0.064	0.433	0.939	1	1	131	17
XGBoost	0.945	0.635	0.389	0.977	0.700	0.500	0.067	0.350	0.829	7	3	129	11

The probability threshold was selected in the development cohort and fixed before temporal validation.

AP, average precision; AUC, area under the receiver operating characteristic curve; FN, false negative; FP, false positive; HL, Hosmer-Lemeshow; TN, true negative; TP, true positive.

**Figure 4:**
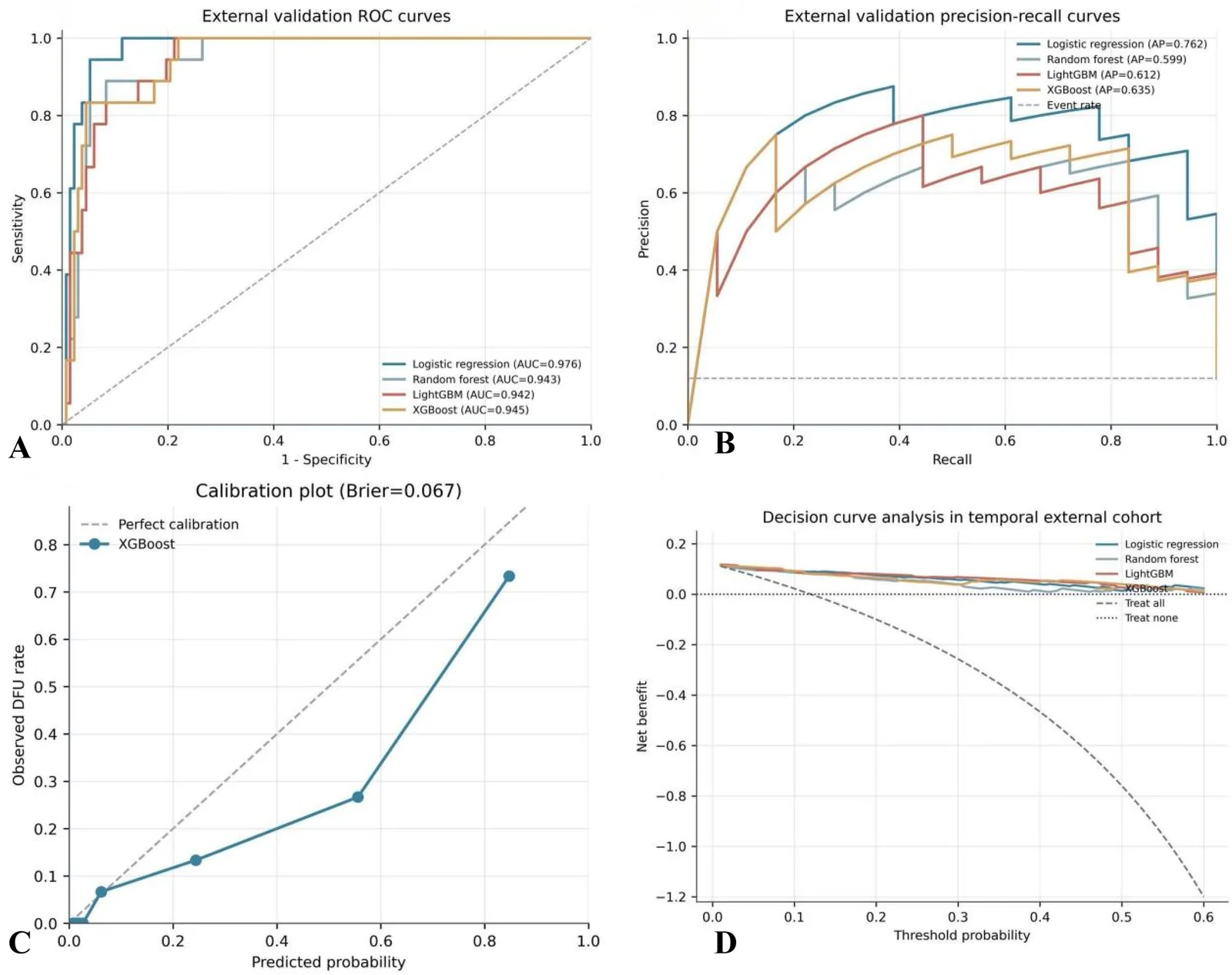
Model performance in the temporal validation cohort. **(A)** Receiver operating characteristic curves; **(B)** precision-recall curves; **(C)** calibration plot for XGBoost; and **(D)** decision curve analysis.

Random forest and XGBoost also demonstrated strong discrimination, with AUCs of 0.943 and 0.945, respectively. Random forest achieved a sensitivity of 0.889, specificity of 0.879, and F1-score of 0.640 at the fixed threshold of 0.472. XGBoost demonstrated a higher specificity of 0.977 but a lower sensitivity of 0.389 at the fixed threshold of 0.829, yielding an F1-score of 0.500. At this threshold, the XGBoost model correctly classified 129 non-DFU patients and seven DFU patients, with three false-positive and 11 false-negative predictions (Figure 5).

**Figure 5:**
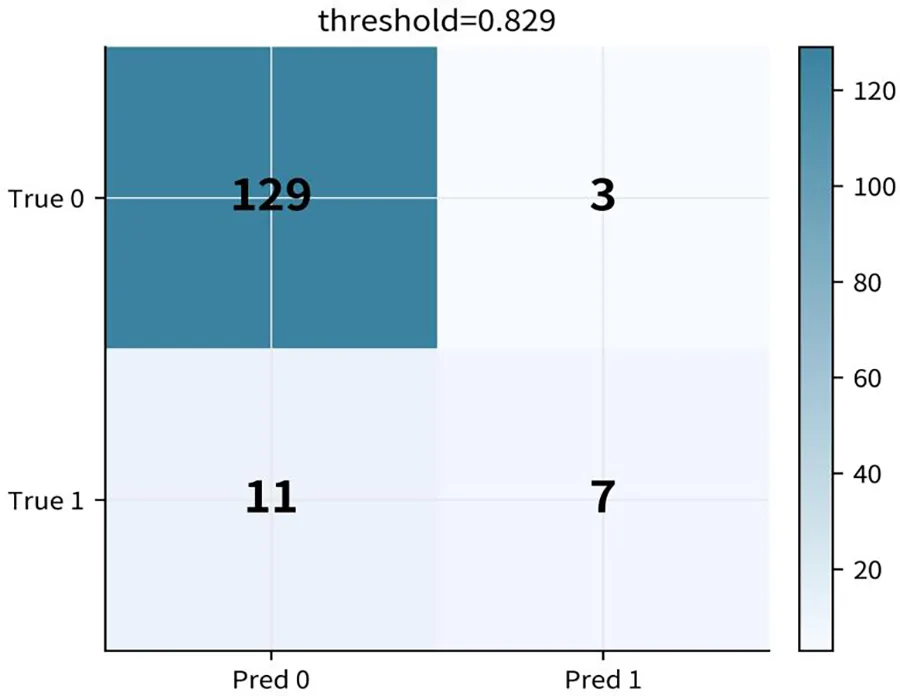
Confusion matrix of the XGBoost model in the temporal validation cohort at the development-cohort threshold of 0.829.

Calibration metrics did not identify a single uniformly superior model. LightGBM had the lowest Brier score (0.064), followed by logistic regression (0.066), XGBoost (0.067), and random forest (0.077). The Hosmer–Lemeshow test indicated evidence of lack of fit for logistic regression (*P* = 0.001), whereas the tests for random forest, LightGBM, and XGBoost were not statistically significant. Accordingly, XGBoost should be described as having favorable rather than superior calibration, because LightGBM had a slightly lower Brier score. Decision curve analysis is shown in Figure 4D.

Threshold selection materially affected the balance between sensitivity and specificity. For screening purposes, a lower threshold may be preferred to prioritize sensitivity, whereas a higher threshold may be appropriate for resource-intensive interventions to reduce false-positive classifications. As an exploratory analysis, optimization by the Youden index in the validation cohort gave logistic-regression sensitivity of 94.4% and specificity of 94.7% at a threshold of approximately 0.542, comparable with the fixed development-cohort threshold (sensitivity 94.4%, specificity 93.2%). Because this alternative threshold was optimized using validation data, it is not externally generalizable. Notably, at the prespecified thresholds, LightGBM and XGBoost sensitivities were only 5.6% and 38.9%, respectively; therefore, neither model should be considered suitable for screening at the current thresholds without prospective threshold recalibration and further external validation.

### 3.4 SHAP interpretation of XGBoost

Mean absolute SHAP values identified ANLR as the most influential XGBoost predictor (1.787), followed by HbA1c (0.900), HALP (0.473), age (0.297), and SII (0.271). The remaining predictors, in descending order, were ABI, diabetes duration, NPAR, neutrophil percentage, CRP, and HDL-C (Table 4). The beeswarm plot showed heterogeneity in the direction and magnitude of predictor contributions across individual patients. Higher HbA1c and SII values generally shifted model predictions toward greater risk, whereas ANLR and HALP displayed more complex patterns. Partial dependence analysis suggested nonlinear relationships between the principal predictors and predicted DFU risk (Figure 6). Because several retained composite indices share source components, the SHAP ranking should be interpreted as model-specific attribution in the presence of correlated features rather than as independent or causal importance.

**Table 4:** XGBoost feature importance ranked by mean absolute SHAP value.

Rank	Predictor	Mean |SHAP|
1	ANLR	1.787
2	HbA1c	0.900
3	HALP	0.473
4	Age	0.297
5	SII	0.271
6	ABI	0.217
7	Diabetes duration	0.199
8	NPAR	0.172
9	Neutrophil percentage	0.110
10	CRP	0.081
11	HDL-C	0.033

SHAP, SHapley additive exPlanations.

Larger values indicate a greater average contribution to the model output.

**Figure 6:**
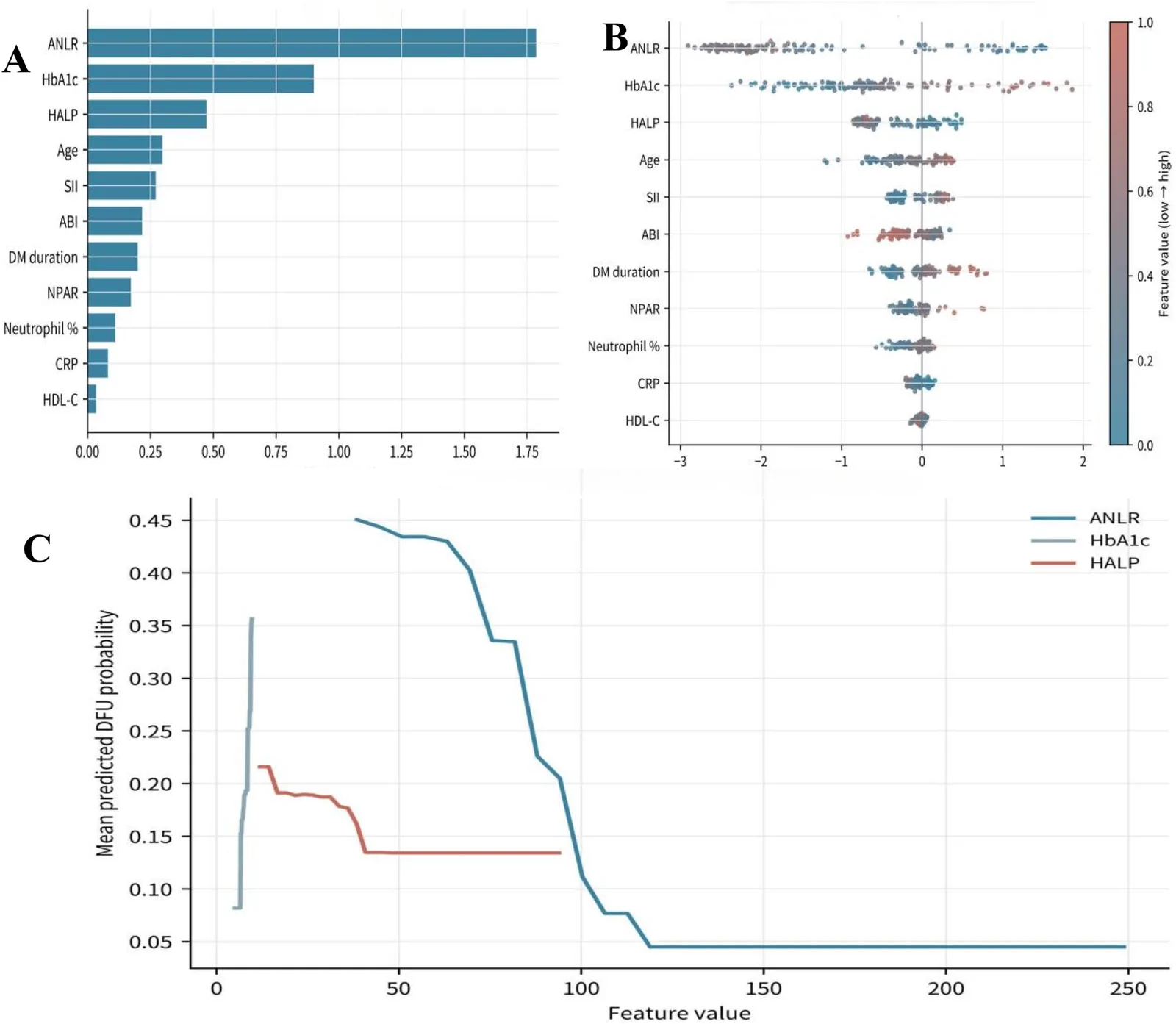
SHAP-based interpretation of the XGBoost model. **(A)** Mean absolute SHAP values; **(B)** SHAP beeswarm plot, with color representing low to high feature values; and **(C)** partial dependence plots for ANLR, HbA1c, and HALP.

## 4 Discussion

Diabetic foot ulcer(DFU) is one of the most severe and destructive chronic complications of diabetes. Approximately 18.6 million diabetic patients worldwide suffer from DFU annually, with a lifetime disease risk ranging from 19% to 34% (19). DFU not only exhibits slow healing and high recurrence rates (up to 65% within 3–5 years) but is also closely associated with infections, amputation, and premature death, with a 5-year mortality rate as high as 30%, and over 70% mortality after amputation (20). Among hospitalized patients, the incidence of DFU ranges from 6% to 16.8%, imposing significant burdens on patients' quality of life, family responsibilities, and healthcare systems. Therefore, early identification of high-risk patients using available diagnostic information during hospitalization constitutes a critical step in proactive DFU prevention. Traditional DFU risk assessments primarily rely on classic risk factors such as disease duration, glycemic control, peripheral vascular disease, and neuropathy (21). Meanwhile, inflammatory-nutritional composite scores derived from complete blood count and biochemical parameters have gained increasing attention due to their cost-effectiveness and accessibility (22). The neutrophil percentage-to-albumin ratio (NPAR) has been demonstrated to positively correlate with DFU risk; the hemoglobin-albumin-lymphocyte-platelet (HALP) score shows a negative correlation with DFU-related amputation risk (22); and the C-reactive protein-to-albumin ratio (CRP/ALB) demonstrates substantial predictive value for DFU-associated amputation (23). These composite indicators comprehensively reflect the host state across three dimensions—immune activation, nutritional status, and systemic inflammation—and may provide additional information for DFU risk prediction that traditional single-indicator approaches cannot achieve. Against this backdrop, this study enrolled hospitalized patients with type 2 diabetes mellitus from 2022 to 2024, developed a model using cases from 2022 to 2023, and conducted time-series external validation with cases from 2024. By combining single-factor screening, LASSO variable compression, multi-model comparison, and SHAP analysis, we constructed and evaluated an in-hospital DFU incidence risk prediction model based on inflammatory-nutritional composite indicators, aiming to provide evidence-based insights for early DFU risk stratification among hospitalized patients.

This study developed a DFU risk prediction model based on 550 hospitalized patients with type 2 diabetes and conducted temporal validation using cases from 2024. The main findings are as follows: (1) routinely available admission data and inflammatory–nutritional composite indices contained information associated with in-hospital DFU risk; (2) logistic regression had the highest observed validation AUC, but its Hosmer–Lemeshow test indicated lack of fit, whereas LightGBM had the lowest Brier score; thus, no model was uniformly superior across discrimination and calibration; (3) ANLR, HbA1c, HALP, age, and SII contributed most strongly to the XGBoost predictions according to SHAP, although these attributions require caution because several predictors share mathematical components; and (4) CRP/ALB did not pass the predefined screening criteria, underscoring that the stability and generalizability of inflammatory–nutritional composite markers require further validation.

Previous studies have indicated that the risk of diabetic foot ulcer (DFU) is closely associated with age, duration of diabetes, poor glycemic control, peripheral vascular disease, infection, and deteriorating nutritional status (7, 24). In this study, HbA1c, arterial stiffness index (ABI), duration of diabetes, and age were included in the final model, consistent with these clinical findings. In recent years, machine learning has been applied to predict DFU occurrence in hospitalized patients, assess DFU amputation risk, and model data from local foot examinations (25, 26). While some studies report that complex models outperform traditional ones, logistic regression achieved the highest area under the curve (AUC) in external validation for this study. This result is not contradictory: given limited sample size, few events, strong predictive signals, and variables that are relatively sparse after LASSO screening, a logistic model with a simple structure and low variance may exhibit superior generalization stability.

This study also supports the potential value of the inflammatory-nutritional composite index in DFU risk assessment. NLR, PLR, SII, CRP/ALB, and HALP have been utilized in recent years to evaluate risks for DFU, diabetic foot infections, amputation, and prognosis (8, 27). ANLR and HALP ranked among the most significant SHAP predictors in this study, suggesting that the combined state of inflammatory response and nutritional status may better reflect hospitalized patients' susceptibility to tissue injury, infection, and healing impairments than individual leukocyte or albumin markers. SII was also included in the final model, indicating that the immune-inflammator*y* axis comprising platelets, neutrophils, and lymphocytes may play a role in DFU risk development. However, ANLR, HALP, SII, and NPAR are derived from overlapping laboratory components, and some source variables were also evaluated or retained. Their apparent importance may therefore reflect shared information, and neither multivariable coefficients nor SHAP values should be interpreted as independent biological effects without formal collinearity and sensitivity analyses.

The clinical value of predictive models cannot be assessed solely by AUC. Logistic regression achieved the highest observed AUC in this study but showed evidence of calibration lack of fit by the Hosmer–Lemeshow test. LightGBM had the lowest Brier score but a sensitivity of only 5.6% at its prespecified threshold, whereas XGBoost had a Brier score of 0.067 and a nonsignificant Hosmer–Lemeshow test but sensitivity of only 38.9%. These findings indicate that no model was uniformly superior across discrimination, calibration, and threshold-dependent classification performance. In particular, LightGBM and XGBoost should not be regarded as screening models at the current fixed thresholds; prospective recalibration and threshold selection are necessary before clinical use. SHAP analysis of XGBoost remains useful for describing how the fitted model distributes risk contributions across predictors, but those explanations are model-specific, may be affected by correlated features, and should not be interpreted as causal effects or as a substitute for clinical judgment. The temporal split by calendar year reduced leakage that can arise from random splitting of highly similar contemporaneous patients, but it does not eliminate other sources of bias or information leakage. TRIPOD + AI and PROBAST + AI emphasize transparent reporting of validation strategies, calibration, and potential biases (28).

## 5 Limitations

This study has several limitations. First, it was a single-center retrospective study. Although a temporal validation cohort was used, geographical external validation was not performed, so generalizability across hospitals, laboratory platforms, and patient populations remains uncertain. Second, the number of DFU events was limited (66 overall, including 48 in the development cohort and only 18 in the temporal validation cohort), which increases the risk of model and threshold instability and limits the precision of performance estimates. Third, ANLR, HALP, SII, and NPAR contain overlapping neutrophil, lymphocyte, albumin, hemoglobin, and platelet information, and some source variables were also considered or retained. Pairwise correlations and LASSO reduce but do not eliminate concerns about redundant information; formal variance-inflation/condition-index diagnostics and sensitivity analyses comparing raw-variable-only and composite-index-only models were not performed. Consequently, individual regression coefficients and SHAP rankings may be unstable or may redistribute importance among correlated predictors. Fourth, the exact interval from admission to ulcer occurrence and hospital length of stay were not modeled, and retrospective outcome ascertainment did not permit verification of blinding to predictor information. Although active DFU at admission was excluded, admission laboratory measurements obtained within 48 h could have been influenced by subclinical or initially unrecognized foot pathology; reverse causation and residual information leakage therefore cannot be excluded. Fifth, established clinical predictors—including prior DFU, peripheral neuropathy severity, foot deformity, plantar pressure, local skin condition or temperature, infection severity, and treatment interventions—were not fully incorporated. Their omission means that the model may partly identify an already high-risk inflammatory, nutritional, or vascular state rather than provide independent early prediction of incident DFU. The model should therefore complement, not replace, structured clinical foot examination and established risk assessment. Sixth, model probabilities were not prospectively recalibrated and no clinical-impact trial was performed; therefore, the current models and thresholds are not ready for direct clinical deployment.

## 6 Conclusion

Using routinely available admission data and inflammatory–nutritional composite indices, the evaluated models showed high observed discrimination for in-hospital DFU risk stratification in this single-center cohort. Logistic regression had the highest observed AUC but showed calibration lack of fit; LightGBM had the lowest Brier score but very low sensitivity at its prespecified threshold; and XGBoost provided model-specific SHAP explanations but was also insufficiently sensitive for screening at the current threshold. Because temporal validation included only 18 DFU events, important established foot-risk variables were omitted, and formal multicollinearity sensitivity analyses were not performed, the findings remain preliminary. Multicenter prospective validation with complete foot-risk phenotyping, threshold and probability recalibration, and clinical-impact assessment is required before clinical use.

## Data Availability

The raw data supporting the conclusions of this article will be made available by the authors, without undue reservation.
